# Association of serum biochemical parameters with growth performance and gut microbiota in large white pigs

**DOI:** 10.3389/fvets.2025.1702154

**Published:** 2026-01-09

**Authors:** Shuang Liang, Yanping Zhang, Qin Xia, Xiaoxiao Liu, Jing Liang

**Affiliations:** College of Animal Science and Technology, Guangxi University, Nanning, China

**Keywords:** serum biochemical parameters, growth performance, gut microbiota, lactate dehydrogenase, large white pigs, serum creatinine

## Abstract

**Background:**

Serum biochemical parameters are crucial indicators of animal health and metabolism, providing valuable insights into nutritional and physiological status.

**Methods:**

To investigate the correlations among serum biochemical indicators, growth traits, and gut microbiota, a total of 274 Large White pigs (124 boars and 150 sows) were selected as experimental subjects in this study. At the early fattening stage (80 days of age), blood samples and fecal samples were collected from all pigs. Five key serum biochemical parameters (Glu, LDH, HDL-C, LDL-C, sCr) and fecal microbial diversity were measured. Additionally, the main economic traits of the pigs were measured following the standard evaluation criteria for swine production performance.

**Results and discussion:**

Results showed that serum creatinine (sCr) was negatively correlated with residual feed intake (RFI), average daily feed intake (ADFI), feed conversion ratio (FCR), and backfat thickness (*r* = −0.13 to −0.25, *p* < 0.05), but positively correlated with loin muscle area (*r* = 0.13, *p* < 0.05). Lactate dehydrogenase (LDH) was negatively correlated with FCR (*r* = −0.24, *p* < 0.05) and RFI (*r* = −0.26, *p* < 0.05). At the genus level, LDH was positively correlated with *Prevotella*, *Faecalibacterium*, *Roseburia*, and *Desulfovibrio*, and negatively correlated with *Fibrobacter*. Meanwhile, sCr showed positive correlations with *Treponema* and *CF231*, and negative correlations with *Subdoligranulum*, *Eubacterium*, and *Dorea*. These genera may serve as microbial biomarkers for sCr and LDH levels. Our findings provide valuable insights for early-stage breeding selection and further research into blood biochemical indicators in pigs.

## 1 Introduction

Serum biochemical parameters are vital indicators of animal health and physiological functions, widely used in clinical diagnostics to assess health status (1, 2). In pig production, serum biochemical markers not only provide effective insights into an animal’s nutritional and metabolic conditions, but also exhibit close associations with economically important traits such as average daily gain, carcass characteristics, residual feed intake (RFI), and feed conversion ratio (FCR) (3–5). RFI and FCR are key metrics used to evaluate feed efficiency in livestock, with RFI serving as an indicator of the difference between actual and expected feed intake, while FCR measures the amount of feed required to produce a unit of weight gain. These measures are crucial for improving production efficiency and reducing feed costs (6, 7). Previous studies have demonstrated the heritability of some biochemical markers, highlighting their utility as indirect selection indicators in genetic improvement (8–10).

Glucose (Glu) serves as a direct and sensitive indicator of energy status and nutrient utilization in pigs, reflecting their overall metabolic condition (11, 12). Low-density lipoprotein cholesterol (LDL-C) and high-density lipoprotein cholesterol (HDL-C) are key regulators of lipid metabolism. In pigs, HDL-C has been shown to influence meat quality by modulating energy metabolism through malate dehydrogenase (MDH) activity in muscle tissue (13, 14). Furthermore, studies in humans have reported that obese individuals exhibit elevated LDL-C and reduced HDL-C levels compared to those of normal weight (15), underscoring the relevance of these lipids in metabolic health. In swine, quantitative trait loci (QTL) mapping and genome-wide association studies have identified genomic regions associated with serum LDL-C and HDL-C concentrations, supporting their utility as biomarkers for fat deposition and metabolic status (16). Serum creatinine (sCr) is widely recognized as a reliable proxy for muscle mass, with strong positive correlations observed with lean body mass (LBM) in various studies (17, 18). Finally, lactate dehydrogenase (LDH) plays a crucial role in systemic metabolism (19), and its serum activity is a key indicator of cellular integrity and tissue health, often serving as an early warning marker for subclinical conditions (20). Recent studies have shown that serum biochemical parameters are influenced not only by genetic factors but also by non-genetic factors—particularly the composition and function of the gut microbiota. Sepp et al. reported a significant association between a decreased proportion of anaerobic microbes in the gut of Jiangquan Black Pig and elevated blood glucose levels and obesity index (21). In addition, an increased abundance of the genus Prevotella was found to effectively improve glucose metabolism in (Long White × Yorkshire × Duroc) (LYD) pigs (22). Furthermore, dietary interventions such as microbial fermented feed have been demonstrated to significantly modulate the serum biochemical profile of finishing pigs, underscoring the dynamic relationship between nutrition, systemic metabolism, and measurable blood parameters (23). Moreover, the genetic background of pigs plays a crucial role in shaping the abundance of specific microbial taxa (24). For instance, in different pig breeds such as Erhualian and Bama miniature pigs, the heritability of certain genera like Ruminococcaceae and Lachnospira can be as high as 0.56, suggesting that gut microbiota are not only regulated by host genetics but may also in turn modulate serum biochemical traits (25). Therefore, shifts in the gut microbial community may serve as potential biomarkers for evaluating production performance and health status in pigs (26).

Serum biochemical parameters hold significant value in reflecting both the production performance and health status of pigs, and the regulatory role of gut microbiota on these indicators should not be overlooked (27). During the fattening stage, feed costs account for approximately 75–85% of total feed cost, making it crucial to investigate the relationships among serum biochemical traits, gut microbiota, and growth performance during this period to enhance economic efficiency (28). However, most prior investigations have either focused on single-dimensional associations or overlooked the fattening stage as a critical window for dissecting these interactions—despite its relevance to feed efficiency and final production outcomes. Based on this context, the present study aimed to analyze the associations between key serum biochemical indicators and production traits in Large White pigs during the fattening stage. Using 16S rRNA gene sequencing, we further identified gut microbial communities that are significantly correlated with specific serum biochemical parameters. Through this research, we seek to provide reliable microbial and metabolic biomarkers for early-stage pig breeding and deepen our understanding of the host–microbiota interactions involved in regulating pig growth performance and health.

## 2 Materials and methods

### 2.1 Animals and husbandry

A total of 274 Large White pigs (124 boars and 150 sows) were selected from a commercial breeding farm in Nanning, Guangxi. The pigs were transferred to the testing station at 65 days of age, underwent a 15-day adaptation period, and were raised until reaching a final body weight of 100 kg. The initial body weight of the pigs, recorded at the commencement of the formal experimental period immediately following the 15-day adaptation phase, was used as the baseline. The initial weight of sows was 23.13 ± 0.83 kg, and that of boars was 24.65 ± 0.59 kg. The farm was equipped with an Osborne automated feeding system and maintained complete growth performance records. All pigs had ad libitum access to feed and water, and were housed under standard conditions with appropriate penning. All swine, irrespective of sex, were provided the same basal diet ad libitum to eliminate dietary variation as a potential confounder in serum or microbiota differences. Nutritional composition of feed is shown in Supplementary Table S1. The experimental farm was equipped with mechanical ventilation (fans) and evaporative cooling (water curtain) systems. Throughout the experimental period, the ambient temperature within the housing facilities was precisely controlled within the range of 18–22 °C. The herd was negative for Pseudorabies virus (PRV), Porcine reproductive and respiratory syndrome virus (PRRSV), Porcine epidemic diarrhea virus (PEDV), Influenza A virus (IAV), *Actinobacillus pleuropneumoniae* (App), and *Mycoplasma hyopneumoniae* (Mhp). Pigs were vaccinated according to standard immunization schedules at the appropriate ages. The sample size required for this study was calculated using G*Power 3.1 software. The statistical parameters employed for the calculation were set as follows: effect size = 0.2, *α* error probability = 0.01, and power (1−*β* error probability) = 0.8.

### 2.2 Sample collection and processing

At 80 days of age, blood samples were collected from the anterior vena cava of each pig using a 20 mL syringe to draw 5 mL of blood, which was transferred into sterile vacuum tubes (124 boars and 150 sows). After allowing the blood to rest at room temperature for 1 h, samples were centrifuged at 3000 rpm for 15 min at 4 °C to isolate the serum. Serum samples were stored at −80 °C until further analysis.

### 2.3 Determination of serum biochemical parameters

Five serum biochemical parameters were measured in all 274 samples: low-density lipoprotein cholesterol (LDL-C), high-density lipoprotein cholesterol (HDL-C), glucose (Glu), serum creatinine (sCr), and lactate dehydrogenase (LDH). Commercial assay kits were purchased from Nanjing Jiancheng Bioengineering Institute (Nanjing, China), and tests were conducted following the manufacturer’s protocols. The specific experimental process is in the Supplementary materials.

### 2.4 Collection and processing of growth performance data

Growth performance data were exported from the Osborne automated feeding system and pig breeding station, including residual feed intake (RFI), average daily feed intake (ADFI), feed conversion ratio (FCR), loin muscle area (LMA), back fat (BF), body length (BL), body height (BH), circumference of cannon bone (CCB), age at 30 kg body weight (30 kg ABW), age at 100 kg body weight (100 kg ABW), and 30–100 kg daily gain (30–100 kg DG). Individuals with missing values for sex, parity, or performance traits were excluded. A new Excel dataset was created to remove outliers based on the criterion of mean ± 3 standard deviations. The cleaned data were saved for subsequent statistical analyses.

### 2.5 16S rRNA gene sequencing and microbiota analysis

All pigs were raised under uniform management conditions. They were fed commercial standard diets and received no antibiotic treatment. At 80 days of age, fecal samples of the 274 pigs were collected directly from the rectum using sterile disposable gloves and placed into autoclaved cryotubes. Samples were immediately flash-frozen in liquid nitrogen and later transferred to −80 °C for storage.

The V3–V4 region of the bacterial 16S rRNA gene was amplified using universal primers 341F (5’-CCTACGGGNGGCWGCAG-3′) and 806R (5’-GACTACHVGGGTATCTAATCC-3′). PCR products were verified by 2% agarose gel electrophoresis and purified using a gel recovery kit (AXYGEN, USA). Sequencing was performed on an Illumina MiSeq platform (Illumina, San Diego, CA, USA) using paired-end mode.

Raw sequencing data were processed using the DADA2 pipeline for primer trimming, quality filtering, denoising, sequence merging, and chimera removal. Taxonomic assignment was conducted using the SILVA (Release 138.2)^1^ with a Naive Bayes classifier and the classify-sklearn algorithm. A phylogenetic tree was constructed using the “qiime phylogeny align-to-tree-mafft-fasttree” workflow, employing MAFFT for sequence alignment and FastTree for tree construction. The resulting ASV (amplicon sequence variant) table was rarefied using the “qiime feature-table rarefy” function to standardize sequencing depth across all samples.

Canonical correspondence analysis (CCA) was applied to evaluate the influence of environmental factors. Seven alpha diversity indices were calculated, including Chao1 and Observed_species (richness), Faith_pd (phylogenetic diversity), Good_coverage (coverage), Shannon and Simpson indices (diversity), and Pielou_e (evenness). Beta diversity was assessed using Bray–Curtis dissimilarity and visualized by principal coordinates analysis (PCoA). The statistical significance of the separation between groups was assessed using permutational multivariate analysis of variance (PERMANOVA) with 999 permutations. Alpha and beta diversity metrics were analyzed using the R packages vegan and ggplot2. To enhance statistical power for detecting associations, the microbiota analysis was performed on individuals selected from the phenotypic extremes of each parameter (the upper and lower 15 individuals for each sex). LEfSe (Linear Discriminant Analysis Effect Size) analysis was performed to identify microbial taxa that were differentially abundant among pre-defined groups. The analysis was conducted across all six taxonomic levels, from phylum to genus. First, the Kruskal–Wallis rank-sum test (*p* < 0.05) was used to identify features with significant differential abundance between groups. Subsequently, Linear Discriminant Analysis (LDA) was employed to estimate the effect size of each significant feature. A threshold LDA score greater than 2.0 was applied for all comparisons to define biologically relevant biomarkers (29).

Biological function prediction was conducted using PICRUSt2^2^ based on the unrarefied ASV table and annotated against the KEGG database.^3^

### 2.6 Statistical analysis

Descriptive statistics and Shapiro–Wilk normality tests for both production traits and serum biochemical indices were performed using SAS 9.4. Pairwise associations between production traits, serum biochemical parameters, and microbial abundance were evaluated using Spearman’s rank correlation. All correlations were tested for significance in R (v4.3.1) via the rcorr() function (Hmisc package), with statistical significance defined as *p* < 0.05. In the description of relevance, p < 0.05 denotes significant correlation, *p* < 0.01 denotes highly significant correlation or extremely significant correlation, and r between 0.2 and 0.4 denotes weak correlation.

## 3 Results

### 3.1 Correlation analysis between serum biochemical parameters and production traits

**Figure 1:**
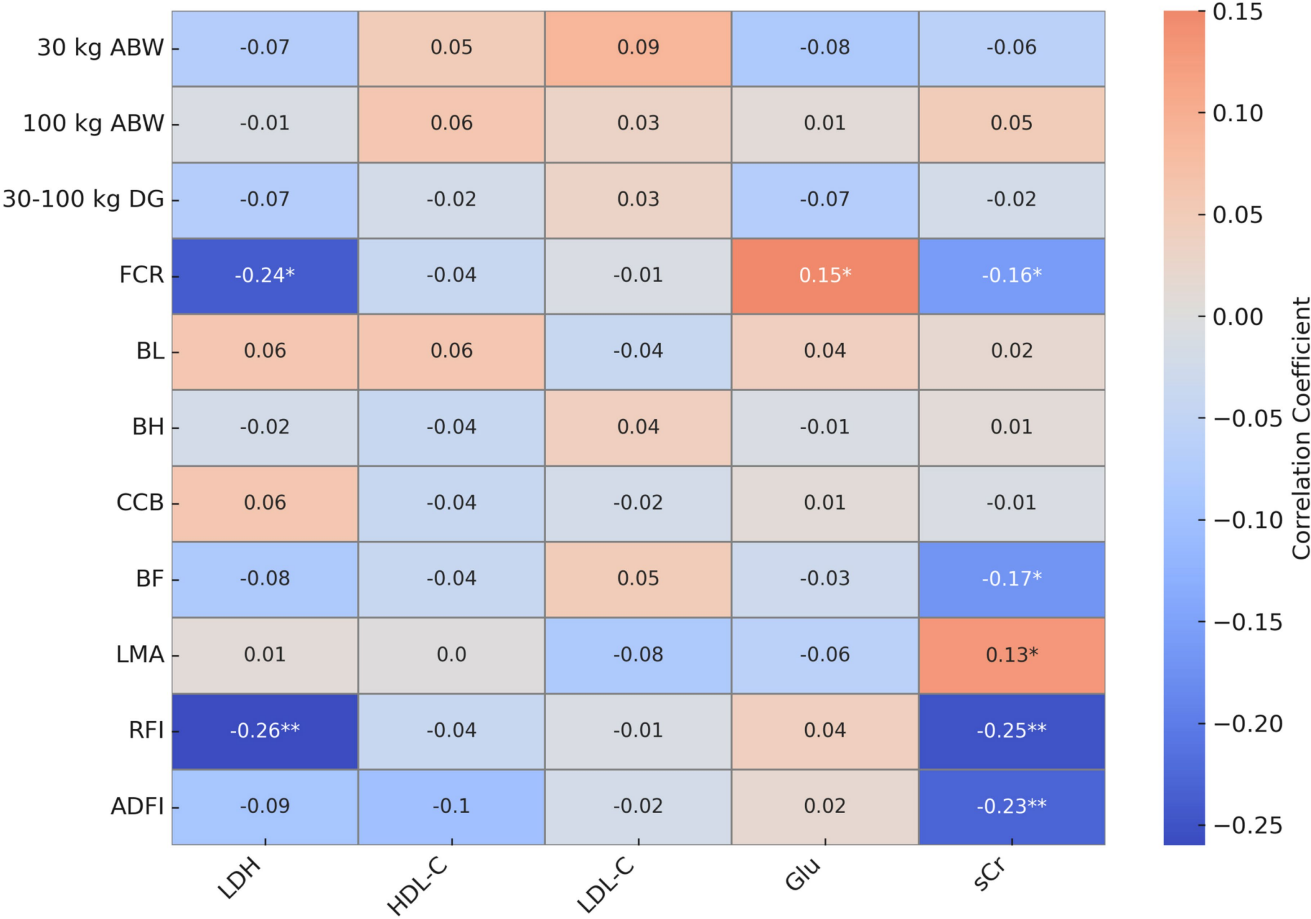
Heatmap of correlation analysis between serum biochemical parameters and production traits. Orange indicates positive correlations, blue indicates negative correlations. *Means significant correlation coefficient (*p* < 0.05), **means extremely significant correlation coefficient (*p* < 0.01).

The correlations between serum biochemical parameters and production traits in Large White pigs (Figure 1). LDH exhibited a weak negative correlations of high statistical significance with FCR (*r* = −0.24, *p* < 0.05), and a weak negative correlation but highly statistical significant with RFI (*r* = −0.26, *p* < 0.01). Glu showed a weak positive association that were statistically significant with FCR (*r* = 0.15, *p* < 0.05). sCr showed significant weak negative correlations despite statistical significance with FCR (*r* = −0.16, *p* < 0.05) and BF (*r* = −0.17, *p* < 0.05), weak negative correlations of extremely statistical significant with RFI (*r* = −0.25, *p* < 0.01) and ADFI (*r* = −0.23, *p* < 0.01), and a weak positive correlations despite statistical significant with LMA (*r* = 0.13, *p* < 0.05). No other serum biochemical parameters showed significant correlations with production traits (*p* > 0.05).

### 3.2 Associations between serum biochemical parameters and gut microbiota

**Figure 2:**
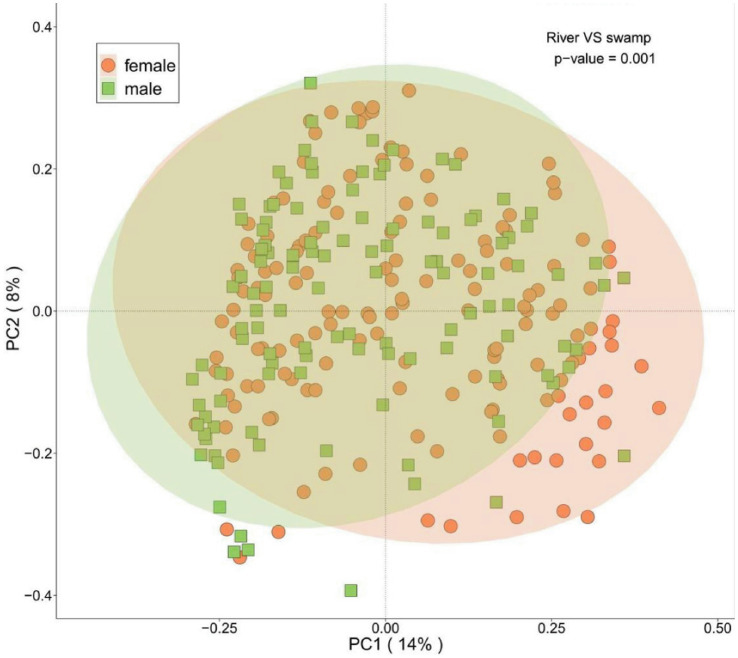
Genus-level gender-specific PCoA analysis. The statistical significance of the separation between groups was assessed using permutational multivariate analysis of variance (PERMANOVA) with 999 permutations (*p* = 0.001). Confidence ellipses (if shown) represent the 95% confidence interval. Orange represents sows (*n* = 150), green represents boars (*n* = 124).

Analysis of ASVs from 274 Large White pigs revealed significant differences in microbial community structure between sexes (Figure 2, *p* = 0.001). To ensure reliable results, we controlled for sex in grouping. For each of the five blood biochemical indicators (LDH, HDL-C, LDL-C, Glu, and sCr), 15 male and 15 female pigs with extremely high or low values were selected and divided into male-high (mh), male-low (ml), female-high (fh), and female-low (fl) groups. Five serum biochemical indicators of extreme individuals of the experimental pigs are summarized in Supplementary Table S2.

**Figure 3:**
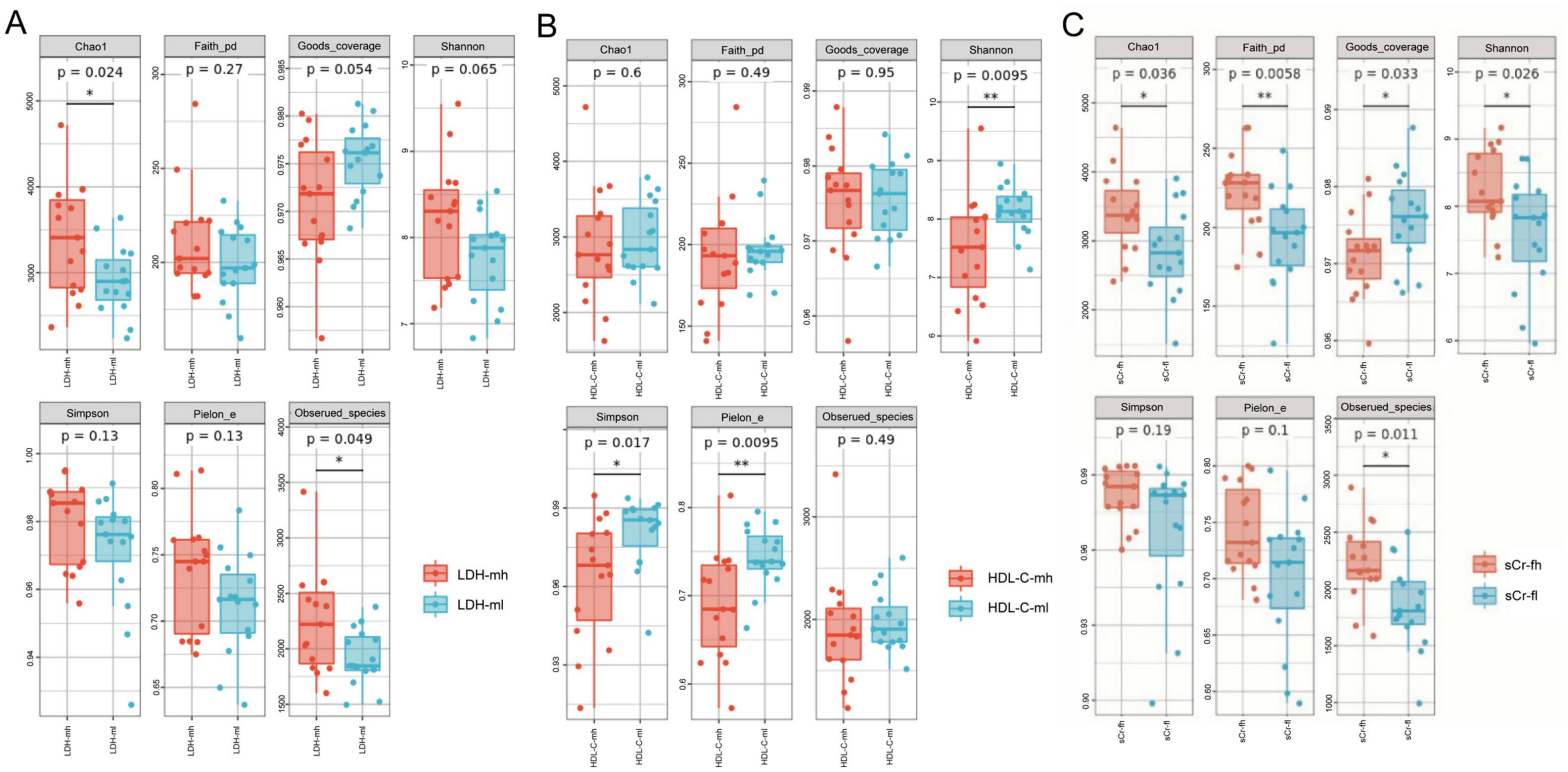
Alpha diversity between high- and low-level groups. Data are depicted in box plots displaying the median, interquartile range, and any outliers. The Mann–Whitney U test was employed to evaluate significant differences between groups, each comprising 15 samples. *Indicates *p* < 0.05, **indicates *p* < 0.01. **(A)** Differences in alpha diversity between groups with high and low LDH in boars. **(B)** Differences in alpha diversity between groups with high and low HDL-C in boars. **(C)** Differences in alpha diversity between groups with high and low sCr of sows.

Alpha diversity analysis showed that in boars, the LDH_mh group had higher microbial richness than the LDH_ml group (Figure 3A). For HDL-C, the ml group had higher richness, diversity, and evenness than the mh group (*p* < 0.01) (Figure 3B). No significant alpha diversity differences were observed for other indicators in boars. In sows, the sCr_fh group exhibited higher species and community richness than the sCr_fl group (Figure 3C), with no significant differences in alpha diversity for other indicators (p > 0.05).

**Figure 4:**
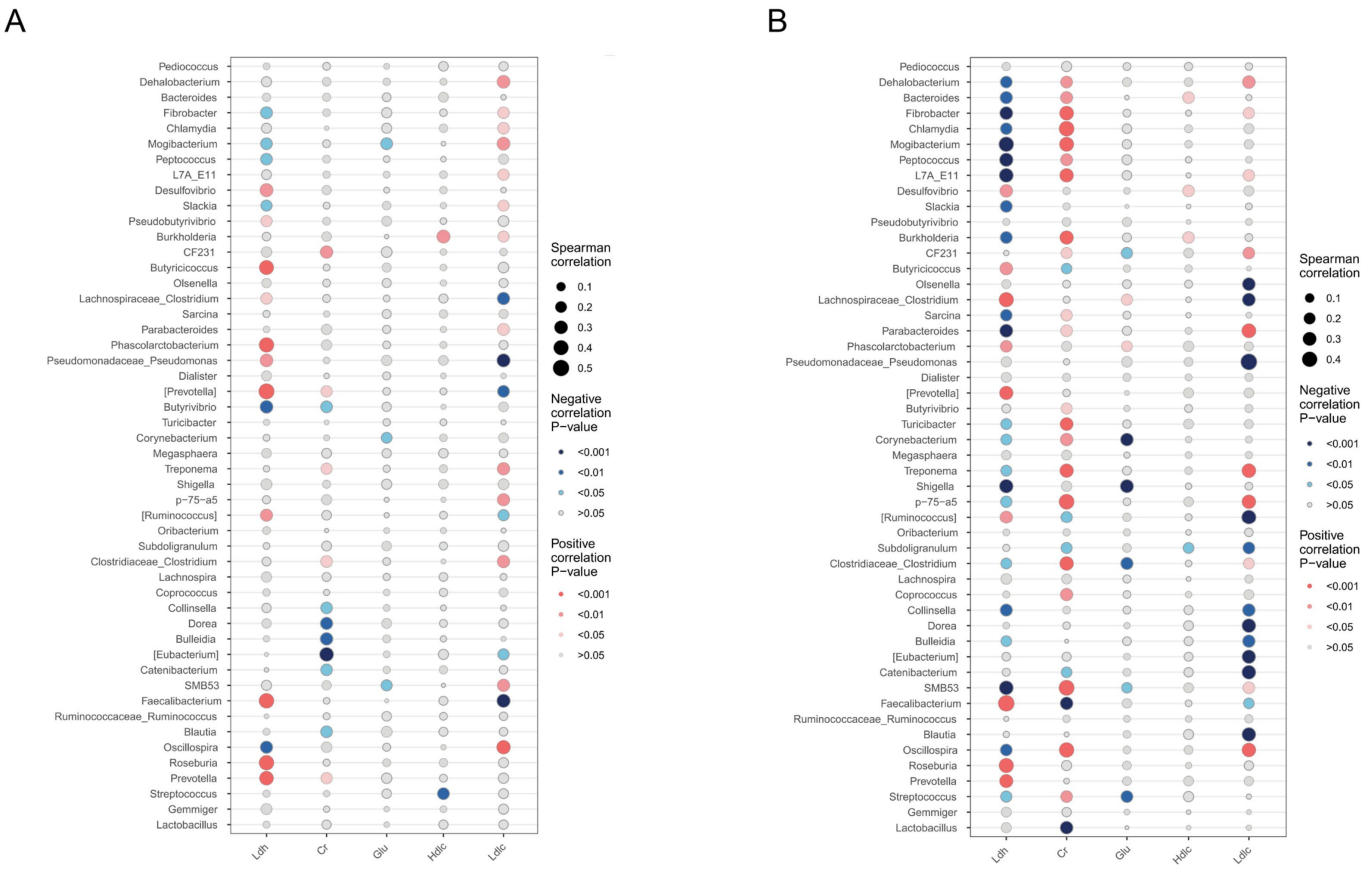
Correlation analysis between different index values and genus-level flora. Red dots indicate a positive correlation between traits and genera (*p* < 0.05), blue dots indicate a negative correlation between traits and genera (*p* < 0.05). The darker the color, the higher the statistical significance (i.e., lower *p* value). The size of the dot indicates the size of the correlation coefficient. The larger the dot, the stronger the correlation.

Spearman correlation analysis was performed between each of the five serum indicators and the top 50 differential genera, stratified by sex to minimize confounding. More genera were found to be significantly correlated in sows than in boars (Figure 4). Genera that were significantly or extremely significantly correlated (*p* < 0.05 or *p* < 0.01) with biochemical parameters in both sexes were summarized in Supplementary Table S3.

Among the genera with consistent trends across sexes, LDH and LDL-C were significantly associated with several genera. For example, *Prevotella*, *Roseburia*, *Faecalibacterium*, and *Butyricicoccus* were extremely positively correlated with LDH (*p* < 0.01), whereas *Oscillospira* and *Fibrobacter* were extremely negatively correlated with LDH (*p* < 0.01). *Treponema*, *Clostridiaceae_Clostridium*, *Turicibacter*, and *CF231* were significantly positively correlated with sCr (*p* < 0.05), while *Catenibacterium* showed a significant negative correlation (*p* < 0.05). Some genera exhibited sex-specific opposite correlation trends. For instance, *Oscillospira*, *Faecalibacterium*, and *Fibrobacter* were negatively correlated with LDH in boars (*p* < 0.05 or *p* < 0.01), but positively correlated with LDL-C in sows (*p* < 0.01).

LEfSe analysis using an LDA score threshold > 2 was performed to identify microbial biomarkers between high and low groups for each indicator. The results regarding boar lactate dehydrogenase are shown in Supplementary Figure S1A, the LDH_mh group was enriched in *Prevotella*, *Roseburia*, *Faecalibacterium*, and *Ruminococcus*, with *Prevotella* showing the highest score; the LDH_ml group was enriched in *Paraeggerthella*. The differential enrichment of microbial taxa between the HDL-C high and low groups is presented in Supplementary Figure S1B, where the HDL-C_mh group was enriched in *Peptostreptococcaceae_Clostridium*, while the HDL-C_ml group was enriched in *Prevotella*. Supplementary Figure S1C illustrates the distinct biomakers for the LDL-C groups, with*Pseudobutyrivibrio* was predominant in the LDL_mh group, whereas the LDL-C_ml group showed enrichment of *Oribacterium* and *Dialister*. Regarding glucose levels (Glu), the microbial biomarkers identified are shown in Supplementary Figure S1D, where the Glu_mh was enriched in *Prevotella*, while Glu_ml was dominated by *Eubacterium*. Finally, the microbial biomarkers associated with serum creatinine (sCr) levels in boars are displayed in Supplementary Figure S1E, *Prevotella* was enriched in the mh group, and *Eubacterium* in the ml group. In sows, the results of the LDH group comparison are depicted in Supplementary Figure S2A, showing that the LDH_fh group was dominated by *Lactobacillus*, while the LDH_fl group was enriched in *SMB53*. Supplementary Figure S2B shows the biomarkers for the HDL-C groups, where the HDL-C_fh group had higher abundance of *Ruminococcus*, and the HDL-C_fl group of *CF231*. For the LDL-C groups, the analysis revealed a distinct enrichment pattern visualized in Supplementary Figure S2C, the LDL-C_fh was enriched in *Butyrivibrio* and *Dialister*, with *Butyrivibrio* scoring highest, whereas the LDL-C_fl showed no significantly enriched genus. The microbial biomarkers differentiating the Glu_fh and Glu_fl groups are presented in Supplementary Figure S2D, with the Glu_fh group was enriched in *Acidaminococcus*, while the Glu_fl group was enriched in *Bifidobacterium*. For sCr, *Oscillospira* was dominant in the fh group, and *Catenibacterium* in the fl group as Supplementary Figure S2E.

**Figure 5:**
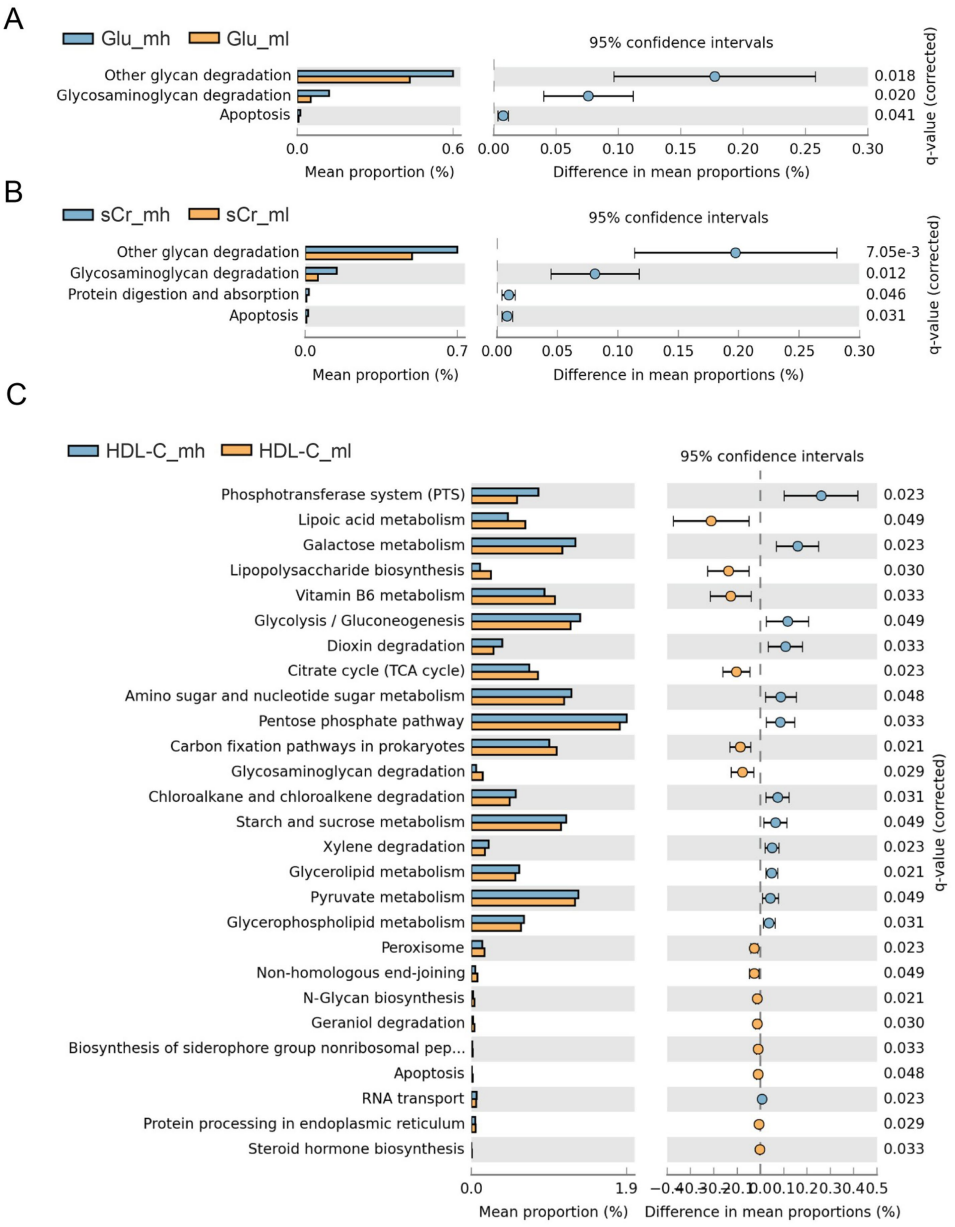
Analysis results for PICRUSt2 in boars. **(A)** Differences in KEGG tertiary pathways of Glu in boars. **(B)** Differences in KEGG tertiary pathways of sCr in boars. **(C)** Differences in KEGG tertiary pathways of HDL-C in boars.

**Figure 6:**
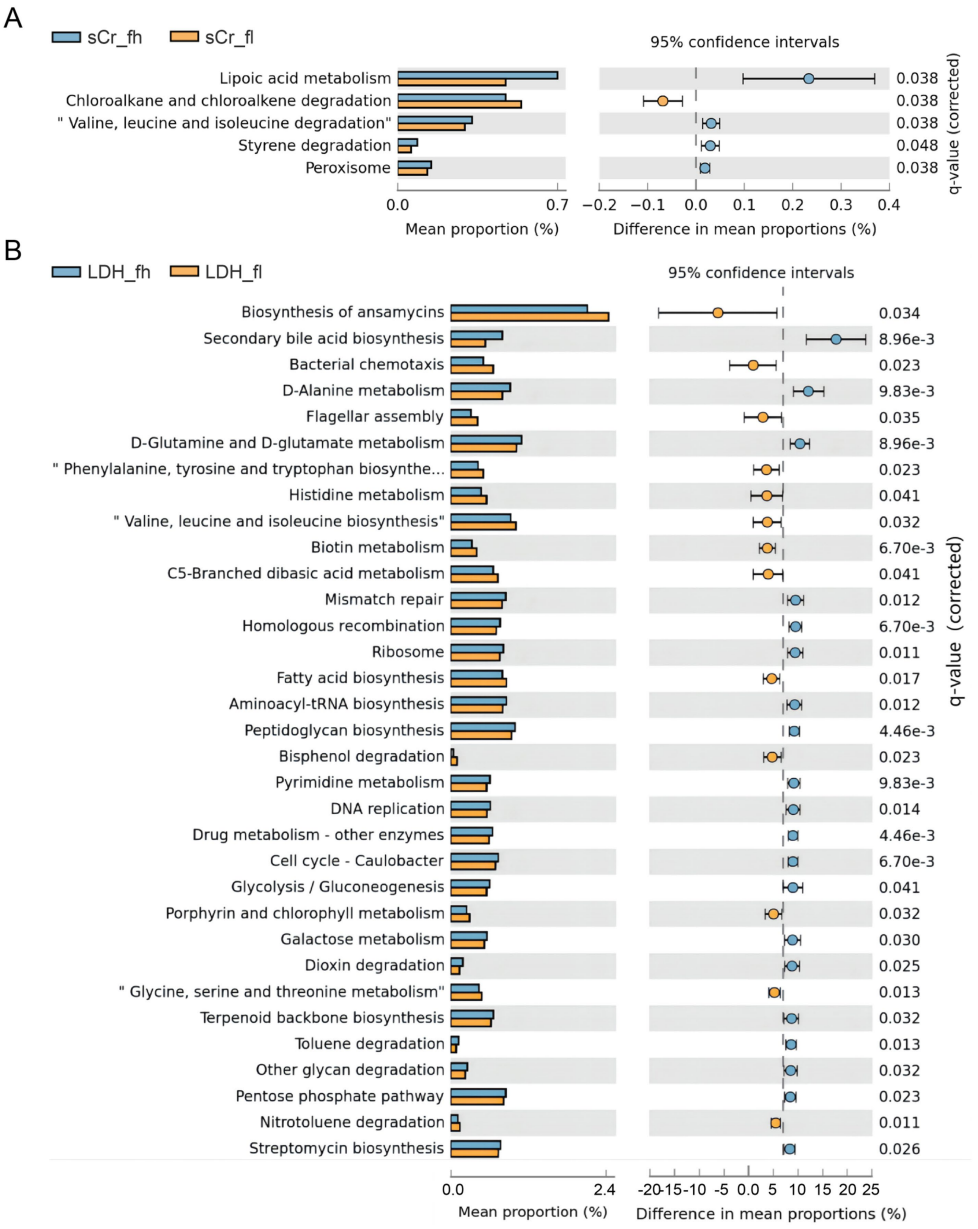
Analysis results for PICRUSt2 in sows. **(A)** Differences in KEGG tertiary pathways of sCr in sows. **(B)** Differences in KEGG tertiary pathways of LDH in sows.

To assess functional differences in gut microbiota between high and low groups, microbial functions were predicted using PICRUSt2. Significant differences in predicted metabolic pathways were observed for three indices in males (Glu, sCr, and HDL-C) and two indices in females (sCr and LDH), while no significant pathways were enriched for the remaining indices (*p* > 0.05). In males, Glu was associated with glycan degradation (Figure 5A); sCr was linked to other glycan degradation and protein digestion and absorption (Figure 5B); HDL-C was enriched for glycolysis/gluconeogenesis and the Tricarboxylic Acid Cycle (TCA cycle) (Figure 5C). In females, sCr was enriched for lipoic acid metabolism (Figure 6A), and LDH was associated with secondary bile acid biosynthesis, D-alanine metabolism, and histidine metabolism (Figure 6B).

## 4 Discussion

Blood physiological and biochemical parameters are important basic indexes of animal growth and development, and are influenced by both genetic and non-genetic factors (30). Due to the easy availability of blood samples, serum biochemical parameters play an important role in predicting growth characteristics and group metabolism and respiratory diseases in pigs (31), which can improve production performance and economic benefits. The gut microbiota is essential for maintaining host morphology and physiological function, particularly in metabolic and immune regulation (32). By conducting a systematic and integrated analysis of serum biochemical parameters, growth traits, and gut microbial communities, the present study identified several serum indicators and microbial biomarkers. These key findings not only provide evidence for implementing early-stage selection strategies in pig breeding programs but also offer informative insights to guide future investigations focused on swine genetic improvement, physiological homeostasis regulation, and the optimization of production performance.

This study found that the absolute values of the correlation coefficients between various biochemical indices and production traits were below 0.3, indicating weak correlations (33). Similarly, some studies also found significant positive correlations between serum Glu, LDL, and TG levels and RFI in Large White pigs, as well as a significant positive correlation between Glu and FCR, with correlation coefficients ranging from 0.146 to 0.197 (34, 35) — consistent with the results of this study. The relatively low correlation coefficients may be due to strong environmental influences and the fact that both serum biochemical traits and host metabolism involve complex, multifactorial processes, where the effect of any single factor tends to be weak.

Interestingly, sCr was significantly correlated with multiple production traits. Specifically, sCr was negatively correlated with backfat thickness, FCR, RFI, and ADFI, but positively correlated with loin muscle area. Kenji et al. found a significant positive correla tion between the cross-sectional area of the psoas major muscle and the serum creatinine-to-cystatin C ratio (CCR) in children with cancer—the larger the muscle area, the higher the CCR (36). Similarly, Cônsolo et al. used metabolomics to compare liver metabolism between cattle with low and high RFI, and found a significant negative correlation between liver creatinine levels and RFI, aligning with our findings (37). Creatinine is one of the key metabolites of muscle metabolism. Exogenous creatinine levels are primarily regulated by nutritional status, as dietary intake of creatine (the precursor of creatinine) directly influences its circulating concentration in the body. In contrast, for endogenous creatinine, apart from pathological factors (renal dysfunction that impairs creatinine excretion), activity level emerges as a major contributor to its fluctuations, particularly in healthy individuals (38). Previous studies have consistently demonstrated that high-intensity or prolonged physical exercise can induce a significant increase in serum creatinine levels, which is attributed to enhanced muscle breakdown and elevated creatine kinase activity (39, 40). Notably, in swine production systems, excessive physical activity has been reported to exert a negative impact on key growth performance indicators, including FCR, RFI, and ADFI (41, 42). Building upon these established concepts, we hypothesize that the negative correlations observed between serum creatinine concentrations and growth performance traits (FCR, RFI, and ADFI) in our study might be partially explained by inter-individual variations in physical activity. Specifically, it is plausible that more active pigs expend more energy, which could contribute to a less efficient feed conversion and concurrently elevate sCr levels due to higher muscle turnover. Consequently, our results suggest that serum creatinine warrants further investigation as a potential biomarker for RFI. However, it is crucial to note that the relationships between serum creatinine, RFI, and ADFI remain poorly explored, and the causal mechanisms underlying the observed correlations are still unclear. Therefore, further research, particularly studies designed to directly measure physical activity and energy expenditure alongside sCr, is needed to validate whether sCr can be used as a reliable marker in genetic selection for feed efficiency.

An increasing number of studies have shown that fecal microbiota transplantation (FMT) can influence pig growth performance, immune function, and serum metabolites (43–46). To investigate which microorganisms can affect pig growth traits and serum biochemical indicators, we performed 16S rRNA gene sequencing on fecal samples. LEfSe analysis showed that the genus *Prevotella* had the highest LDA score in the Glu and LDH high-value groups of male pigs. Studies have shown that *Prevotella* can alter the composition and function of the ecosystem, leading to a reduction in short-chain fatty acids (particularly acetate), which in turn decreases intestinal IL-18 levels during homeostasis and triggers intestinal inflammation (47–49). This inflammation causes damage and rupture of intestinal epithelial cells, resulting in the rapid release of intracellular lactate dehydrogenase (LDH) and an increase in serum LDH levels (50). Additionally, intestinal inflammation impairs the absorption of nutrients and reduces feed conversion efficiency, which may explain the negative correlation observed between LDH and feed conversion ratio (FCR) as well as residual feed intake (RFI). The genus *Ruminococcus* was significantly enriched in both male and female pigs with high HDL-C levels. Ji et al. conducted a 12-week intervention study on hyperlipidemic rats and found that fermented red raspberry treatment significantly reduced body weight, total cholesterol (TC), triglycerides (TG), and LDL-C, while increasing HDL-C levels (51). Their microbiome analysis also showed a reduction in the Firmicutes-to-Bacteroidetes ratio and an increase in the abundance of bacteria such as *Prevotella* and *Ruminococcus*.

The genus *Eubacterium* was significantly enriched in the sCr low-value groups of both male and female pigs. E*ubacterium* can utilize dietary fiber for fermentation, producing short-chain fatty acids (SCFAs) such as butyrate and acetate (52–54). These metabolites exert multiple physiological effects: they help reduce intestinal inflammation and enhance the integrity of the gut barrier, while also serving as an additional energy source for the host (55). This may be one of the key mechanisms by which they improve feed conversion ratio (FCR) and residual feed intake (RFI). However, there is currently insufficient evidence to support a direct correlation between serum creatinine (sCr) and feed efficiency, and their relationship requires further experimental investigation. *Pseudobutyrivibrio* achieved the highest LDA score in the LEfSe analysis associated with low-density lipoprotein cholesterol (LDL-C), indicating its significance as a biomarker in the LDL-C differential group. This bacterium is extensively involved in the metabolism of carbohydrates, proteins, and lipids (56–58). Its major metabolite, butyrate, has been demonstrated in multiple studies as an effective agent for reducing blood cholesterol levels (59). Given that LDL-C primarily functions to transport cholesterol in the bloodstream, the ability of Pseudobutyrivibrioto modulate cholesterol metabolism via butyrate production may explain why it was identified as a top-ranked microbe in the LDL-C-based LEfSe analysis.

The KEGG functional analysis of gut microbiota revealed a predominant enrichment in metabolic pathways. Specifically, glycan degradation, glycosaminoglycan degradation, and apoptosis pathways were significantly enriched in the boar groups with high levels of Glu and sCr. Our results showed that the relative abundance of the phylum Bacteroidetes was higher in the high Glu and sCr groups compared to the low groups. Bacteroidetes are capable of binding polysaccharides via surface glycan-binding proteins (SGBPs), which are then partially degraded by surface glycan-degrading enzymes (60). The resulting oligosaccharides are subsequently transported into the periplasmic space through adjacent gene pairs encoding SusCH (TonB-dependent outer membrane transport proteins) and SusDH (associated glycan-binding proteins), where they undergo enzymatic degradation into monosaccharides (61). These monosaccharides are then transported into the cytoplasm via inner membrane transporters, completing the utilization of polysaccharides (62).

Glycerolipid metabolism and glycerophospholipid metabolism were significantly enriched in boars with high HDL-C levels, which may be related to the high phospholipid content of HDL-C. The regulation of glycerolipid biosynthesis is critical for lipid storage and membrane homeostasis in cells (63). Glycolysis/gluconeogenesis pathways were significantly enriched in sows with high LDH levels. LDH is one of the key enzymes involved in anaerobic glycolysis and gluconeogenesis, suggesting that gut microbiota may influence LDH levels. This finding is consistent with previous research, which demonstrated that gut microbes contribute to explaining variation in porcine serum lipid indicators (64). Several studies have suggested that carbohydrate and lipid metabolic pathways in bacteria are often enriched in pigs with better growth performance and feed efficiency (65). In our study, LDH was significantly negatively correlated with FCR, in agreement with previous findings.

The significant difference in Ruminococcus abundance between HDL-C extreme groups, which did not correlate strongly across the entire population, suggests a non-linear or threshold relationship rather than a contradiction. This association may be particularly strong or biologically relevant at the extremes of the HDL-C distribution, while weaker or masked in the middle due to confounding factors. Therefore, we exercise greater caution in interpreting these results and view the identified associations, such as with Ruminococcus, as potential biomarkers that require further validation in larger, more mechanistic studies.

This study reveals significant correlations between specific serum biomarkers, such as sCr and LDH, and key growth traits like RFI, FCR, and loin muscle area in Large White pigs, suggesting their potential as early indicators of growth performance. Additionally, we identified microbial genera, including Prevotella, Eubacterium, Subdoligranulum, and Ruminococcus, that are significantly associated with serum biochemical parameters and may serve as candidate microbial markers. These findings highlight the potential for integrating both serum and microbiome profiles into breeding programs to improve feed efficiency, growth rates, and overall productivity in pigs. However, the primary limitation of this study lies in its observational design, which inherently limits our ability to establish causal relationships and makes the results susceptible to confounding by unmeasured variables. While several serum biochemical parameters (e.g., sCr, LDH) and gut microbial taxa (e.g., *Subdoligranulum*, *Eubacterium*, *Dorea*) were identified as statistically significant, albeit weak, correlates of growth traits in Large White pigs, these associations should be interpreted with caution. The modest correlation strengths likely reflect the complex, polygenic, and multifactorial nature of growth traits, influenced by genetic, nutritional, management, and environmental factors. Additionally, the study population was limited to a single farm, which may restrict the generalizability of the findings. The observed correlations suggest potential biomarkers but do not establish causal relationships, as the lack of experimental validation prevents determining whether the associations are causal or incidental. Given the weak effect sizes, the predictive value of any individual parameter for breeding applications appears limited. Future studies should therefore focus on two complementary directions: validating these correlative findings in larger and more diverse populations, and implementing interventional experiments—such as probiotic supplementation or targeted dietary modifications—to rigorously test the hypothesized mechanisms linking specific microbes to host physiology. Such investigations are essential to confirm the observed relationships and evaluate their practical relevance for enhancing growth traits in swine.

## 5 Conclusion

In this study, we systematically explored the associations among serum biochemical parameters, growth performance, and gut microbiota composition in Large White pigs, identified several serum indicators and microbial biomarkers. Serum and microbiome samples were collected at early stage of the finishing period (80 days of age), to facilitate the early detection of physiological markers predictive of growth performance outcomes at market age. Our results revealed that specific biochemical indicators, such as sCr and LDH, were significantly correlated with key production traits including RFI, FCR, and loin muscle area, suggesting their potential as early biomarkers for growth performance. Additionally, 16S rRNA gene sequencing identified specific microbial members associated with these biochemical parameters. Notably, genera including *Prevotella* and *Roseburia* were positively correlated with LDH, whereas *Eubacterium* was negatively correlated with sCr, and *Oscillospira* was positively correlated with LDL-C. These findings contribute to the identification of metabolic and microbial markers for pig breeding and provide new perspectives on the host–microbiota interaction.

## Data Availability

The original contributions presented in the study are publicly available. Metagenomic sequencing data pertaining to this paper have been deposited in the NCBI SRA database, accession number: PRJNA1381524.

## Footnotes

1
https://www.arbsilva.de/news/view/2024/07/11/silva-release-1382/

2
https://github.com/picrust/picrust2/wiki

3
https://www.kegg.jp/
