# Supplementary figures and images for: Association of serum biochemical parameters with growth performance and gut microbiota in large white pigs
Source: Front Vet Sci. 2026 Jan 9;12:1702154. doi: 10.3389/fvets.2025.1702154 (PMC12827089; doi:10.3389/fvets.2025.1702154)

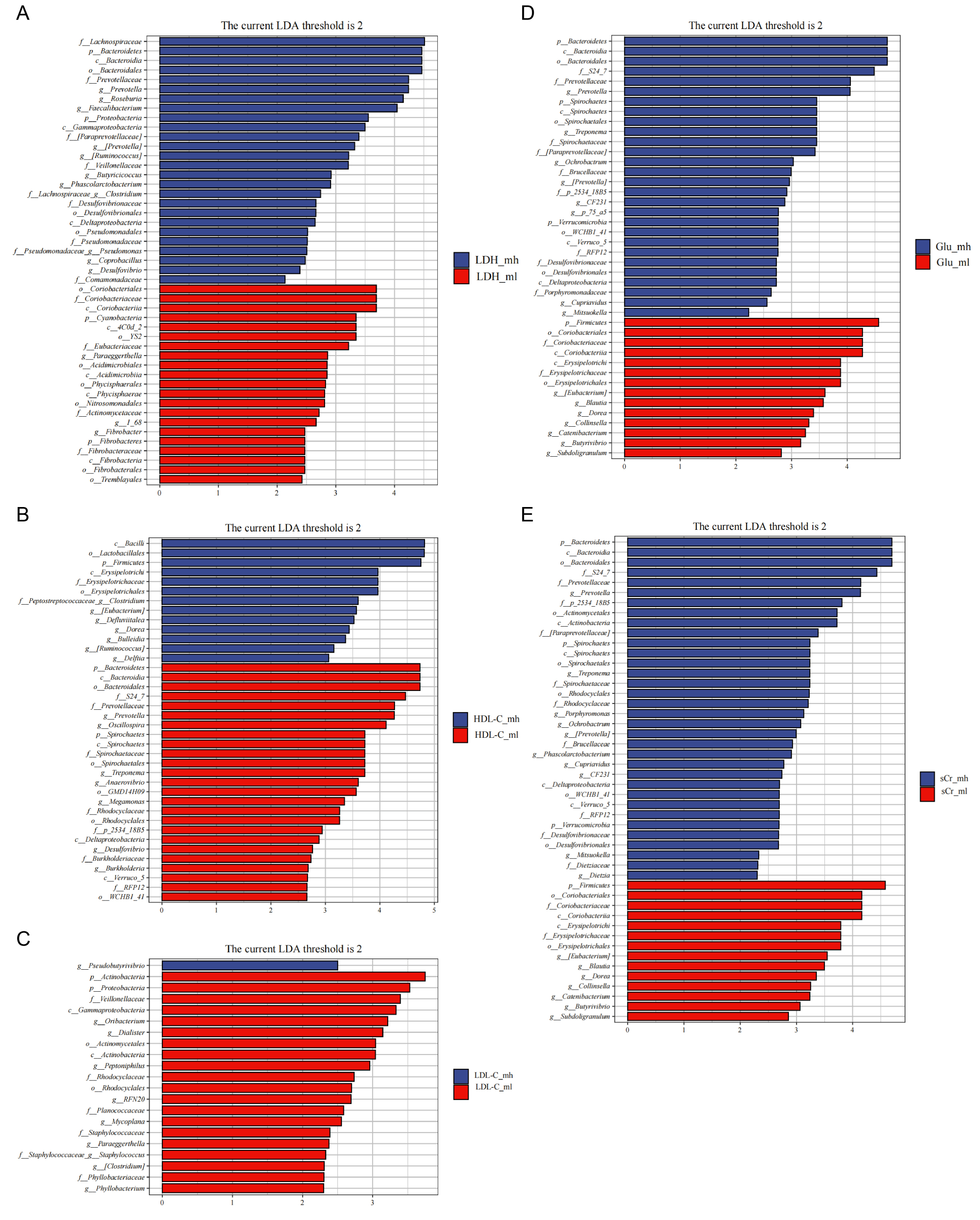

Supplement: Supplementary file 1 [file Image_1.jpeg]

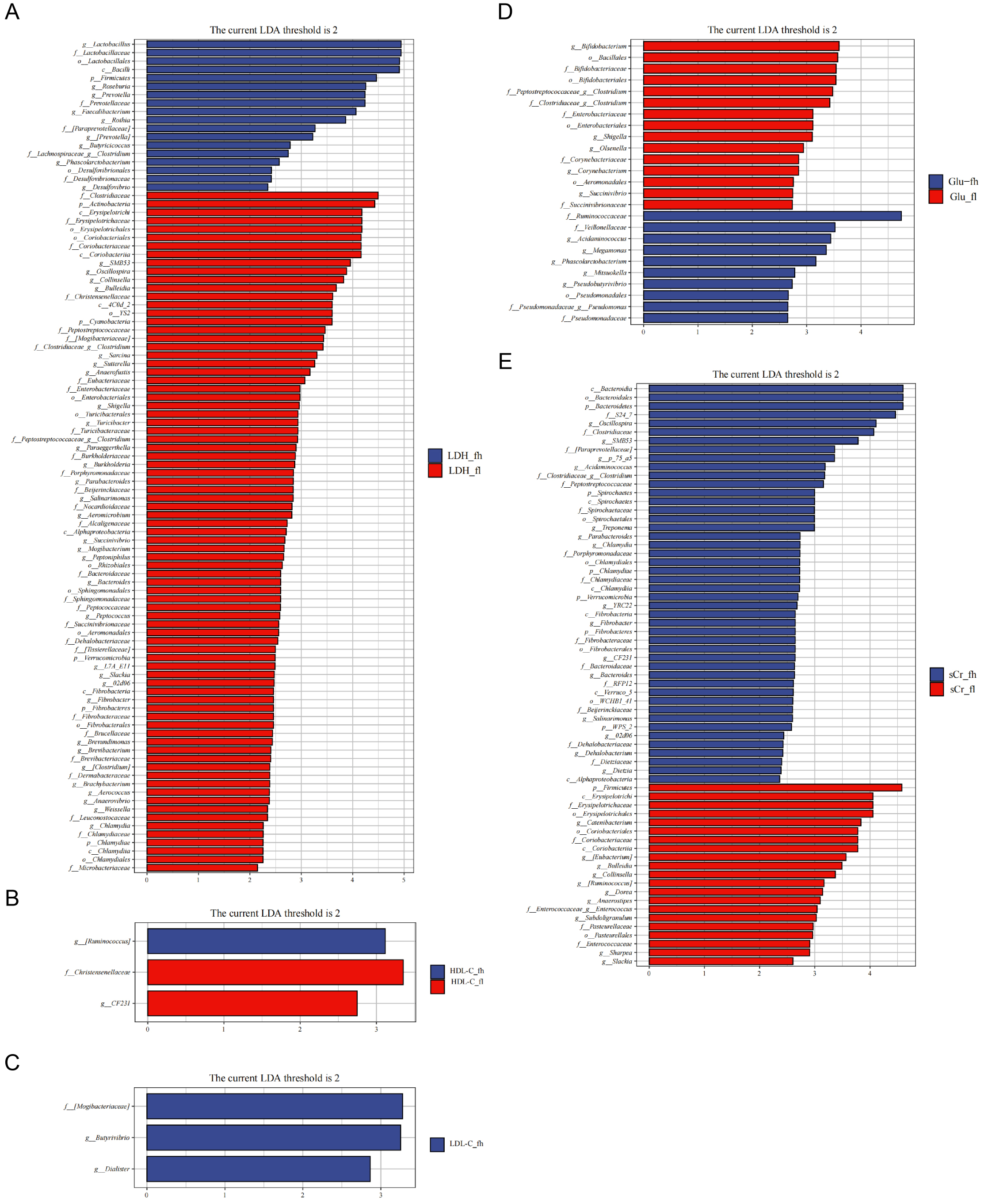

Supplement: Supplementary file 2 [file Image_2.jpeg]
