# Supplementary material for: Association of serum biochemical parameters with growth performance and gut microbiota in large white pigs
Source: Front Vet Sci. 2026 Jan 9;12:1702154. doi: 10.3389/fvets.2025.1702154 (PMC12827089; doi:10.3389/fvets.2025.1702154)
Supplement: Supplementary file 3 [file Table_1.pdf]

Table S1. Nutritional Composition of the Formulated Pig Feed

Nutrient	Content	Unit
Crude protein	12.5–15.5	%
Crude fiber	⩽5.0	%
Crude ash	⩽8.0	%
Calcium (Ca)	0.5–1.2	%
Total phosphorus (P)	0.3–0.9	%
Sodium chloride (NaCl)	0.3–1.2	%
Lysine	⩾0.9	%

Basal diet was formulated according to the nutritional requirements of the National Research Council (NRC, 2012).

Table S2. Statistical analysis of differences between high and low groups for five serum biochemical indicators in Large White Pigs

Serum biochemical parameters	m_h (N=15)	m_l (N=15)	***P*** Value	f_h (N=15)	f_l (N=15)	***P*** Value
LDH	3309.55±199.53	1705.95±158.45	5.37E-20	3411.17±127.05	1602.16±274.84	2.15E-19
SCR	82.48±3.57	47.71±3.68	7.38E-21	84.80±3.44	50.06±3.10	4.78E-22
GLU	7.07±0.34	3.58±0.52	1.13E-18	7.61±0.40	3.84±0.44	4.39E-20
HDL-C	0.86±0.05	0.47±0.05	3.47E-19	0.96±0.03	0.50±0.44	7.50E-22
LDL-C	1.33±0.08	0.61±0.06	1.11E-21	1.31±0.07	0.60±0.44	5.43E-25

Note: m_h = high-value males; m_l = low-value males; f_h = high-value females; f_l = low-value females.

Table S3. Summary of Genus-Level Microbial Correlations with Serum Biochemical Parameters in Large White Pigs

Indicator	Lactate dehydrogenase （LDH）	Serum creatinine （sCr）	Glucose （GLU）	High density Lipoprotein cholesterol （HDL-C）	Low density Lipoprotein cholesterol （LDL-C）
Positively correlated bacterial genus	*Prevotella*、<br>*Roseburia*<br>*Faecalibacterium*<br>*Phascolarctobacterium*<br>*Lachnospiraceae_C*	*Treponema*<br>*Clostridiace*		*Burkholderia*	*Oscillospira*<br>*SMB53*<br>*p-75-a5*<br>*Treponema*、

	lostridium	*ae_Clostridium*		*Parabacteroides*
	Butyricicoccus、*Ruminococcus*	*Turicibacter*		*L7A_E11*、*Fibrobacter*、
	Desulfovibrio *Oscillospira*	*CF231*		*Dehalobacterium* *Faecalibacterium*
Negatively correlated bacterial genus	*Slackia*	*Catenibacterium*	*SMB53*	*Eubacterium*
	Mogibacterium			*Lachnospiraceae_Clostridium*
	Corynebacterium		*Corynebacterium*	*Pseudomonadaceae_Pseudomonas*
	Fibrobacter			

File 1. Protocol for Serum Biochemical Parameter Analysis

Specific Determination Procedure for Lactate Dehydrogenase (LDH) (Microplate Method):

Product Batch Number：A020-1.

Prior to the assay, serially dilute the pyruvate standard (2 μmol/mL) with double-distilled water to 1/200, 1/100, 1/50, 1/20, 1/10, 1/5, and 1/2 of the original concentration to prepare the standard curve.

(1) Store the kit at 4℃. Before the start of the experiment, equilibrate it at room temperature for 20 min.

(2) Add double-distilled water: add 25 μL to the blank well; add 5 μL to the standard well and the control well respectively; do not add to the determination well.

(3) Sequentially add 20 μL of pyruvate standards with each concentration gradient to the standard wells; add 20 μL of the serum sample to be tested to the determination well and the control well respectively.

(4) Add 25 μL of reaction buffer to the blank well, standard well, determination well, and control well.

(5) Add 5 μL of coenzyme Ⅰ working solution to the determination well, mix well, and incubate in a constant - temperature incubator at 37℃ for 15 min.

(6) Add 25 μL of 2,4-dinitrophenylhydrazine to the blank well, standard well, determination well, and control well, mix well, and incubate in a constant - temperature incubator at 37℃ for 15 min.

(7) Add 250 μL of 0.4 M NaOH solution to the blank well, standard well, determination well, and control well.

(8) Mix well, let stand at room temperature for 5 min, and then use a microplate reader to determine the absorbance value (wavelength 450 nm).

(9) Plot the measured absorbance on the y-axis against the corresponding concentration on the x-axis to generate the standard curve.

$$\text{Activity of LDH in serum (plasma) (U/L)} = \frac{\text{OD value of determination} - \text{OD value of control}}{\text{OD value of standard} - \text{OD value of blank}} \times \text{Concentration of standard (0.2 μmol/mL)} \times N \times 1000$$

Specific Determination Procedure for Creatinine (sCr) (Microplate Method):

Product Batch Number：C011-2.

(1) Store the kit at 4℃. Before starting the experiment, equilibrate it at room temperature for 20 min to ensure the stability of reagents.

(2) Sample/Standard Addition: To the standard wells: add 6 μL of creatinine standards (at respective concentration gradients). To the determination wells: add 6 μL of serum samples to be tested. To the blank wells: add 6 μL of double-distilled water.

(3) Enzyme Solution A Addition: Add 180 μL of Enzyme Solution A to the blank wells, standard wells, and determination wells respectively.

(4) First Incubation and A_1 Measurement: Incubate the microplate in a constant - temperature incubator at 37℃ for 5 min. Then, use a microplate reader to measure the absorbance value (A_1) at a wavelength of 546 nm.

(5) Enzyme Solution B Addition: Add 60 μL of Enzyme Solution B to the blank wells, standard wells, and determination wells respectively.

(6) Second Incubation and A_2 Measurement: Incubate again in a constant - temperature incubator at 37℃ for 5 min. Then, measure the absorbance value (A_2) at a wavelength of 546 nm using a microplate reader.

$$K = \frac{\text{Sample Volume} + \text{Volume of Enzyme Solution A}}{\text{Sample Volume} + \text{Volume of Enzyme Solution A} + \text{Volume of Enzyme Solution B}} = \frac{186}{246}$$

$$\text{Creatinine Concentration } (\mu\text{mol/L}) = \frac{(\text{Determination } A_2 - K \times \text{Determination } A_1) - (\text{Blank } A_2 - K \times \text{Blank } A_1)}{(\text{Standard } A_2 - K \times \text{Standard } A_1) - (\text{Blank } A_2 - K \times \text{Blank } A_1)} \times \text{Concentration of Creatinine Standard}$$

Specific Determination Procedure for Glucose (Glu):

Product Batch Number：F006-1-1.

(1) Store the kit at 4℃. Before the experiment begins, equilibrate it at room temperature for 20 min to ensure reagent stability.

(2) Sample/Calibrator/Blank Addition: To blank wells: add 3 μL of distilled water. To calibration wells: add 3 μL of the calibration standard (5.55 mM). To sample wells: add 3 μL of the samples to be tested.

(3) Reagent Addition: Add 300 μL of the glucose determination reagent to blank wells, calibration wells, and sample wells, respectively.

(4) Incubation and Absorbance Measurement: Gently shake the microplate to mix the contents well. Incubate in a constant - temperature incubator at 37℃ for 15 min. Then, use a microplate reader to measure the absorbance value A at a wavelength of 505 nm.

$$\text{Glu Concentration (mmol/L)} = \frac{A_{\text{determination}} - A_{\text{blank}}}{A_{\text{calibration}} - A_{\text{blank}}} \times C_{\text{calibration}}$$

Specific Determination Procedure for High - Density Lipoprotein Cholesterol (HDL-C and LDL-C):

Product Batch Number：A112-1(HDL-C)、A113-2(LDL-C).

(1) Store the kit at 4℃. Before the experiment, equilibrate it at room temperature for 20 min to ensure the stability of reagents.

(2) Sample/Calibrator/Blank Addition: To blank wells: add 2.5 μL of distilled water. To calibration wells: add 2.5 μL of the calibrator. To sample wells: add 2.5 μL of the samples to be tested.

(3) R1 Working Solution Addition: Add 180 μL of R1 working solution to blank wells, calibration wells, and sample wells, respectively.

(4) First Incubation and A_1 Measurement: Gently shake to mix well. Incubate in a constant - temperature incubator at 37℃ for 5 min. Then, use a microplate reader to measure the absorbance value (A_1) of each well at a wavelength of 546 nm.

(5) R2 Working Solution Addition: Add 60 μL of R2 working solution to blank wells, calibration wells, and sample wells, respectively.

(6) Second Incubation and A_2 Measurement: Gently shake to mix well. Incubate in a constant - temperature incubator at 37℃ for 5 min. Then, use a microplate reader to measure the absorbance value (A_2) of each well at a wavelength of 546 nm.

$$\text{HDL-C Concentration (mmol/L)} = \frac{(\text{Sample } A_{\text{determination2}} - \text{Sample } A_{\text{determination1}}) - (\text{Blank } A_2 - \text{Blank } A_1)}{(\text{Calibration } A_2 - \text{Calibration } A_1) - (\text{Blank } A_2 - \text{Blank } A_1)} \times C_{\text{calibration (mmol/L)}}$$
